# 4182 Use of Machine Learning for Surgical Outcomes Research: what are the clinical implications?

**DOI:** 10.1017/cts.2020.187

**Published:** 2020-07-29

**Authors:** Priscila Rodrigues Armijo, Sindhura Bonthu, Alicia Schiller, Qiuming Zhu, Tiffany Tanner

**Affiliations:** 1 University of Nebraska Medical Center - Great Plains IDeA-CTR; 2 University of Nebraska - Omaha; 3 University of Nebraska Medical Center

## Abstract

OBJECTIVES/GOALS: Multivariate regression is used for surgical outcomes analyses; but does not allow for evaluation of all variables. Machine learning could be the perfect alternative to address this issue. Our aim was to evaluate whether machine learning is a feasible alternative to evaluate surgical outcomes. METHODS/STUDY POPULATION: H-CUP National Inpatient Sample database was queried for adult patients with colorectal cancer who underwent colorectal resection, while the NSQIP database was queried for adult patients with rectal cancer who underwent proctectomy. A multivariate regression analysis was performed to assess risk factors associated with 30-day complications following those procedures. Subsequently, machine learning techniques of under-sampling and oversampling were applied to the same datasets for the evaluation of risk factors for the same outcome. These techniques were used to achieve a larger population sample size and to detect statistical significance. Results between the two methodologies were compared. RESULTS/ANTICIPATED RESULTS: Multivariate regression revealed that open approach, gender, race, geographic location, number of comorbidities, and type of insurance was associated with increased 30-day mortality in colorectal resection patients. Conversely, the use of machine learning revealed that preoperative weight loss, preexistent chest heart failure, renal failure or perivascular disease were strongly associated with 30-day mortality. For proctectomy patients, multivariate regression found no association between surgical approach and 30-day mortality. However, machine learning revealed gender, hypertension, and reoperation to be strongly associated with 30-day mortality. DISCUSSION/SIGNIFICANCE OF IMPACT: Machine learning enabled multiple combinations that were not possible to examine in a conventional multivariate regression analysis. Machine learning compared to traditional multivariate regression produced significantly different outcomes, highlighting the need for in depth of these methodologies.

